# Tat-Cannabinoid Receptor Interacting Protein Reduces Ischemia-Induced Neuronal Damage and Its Possible Relationship with 14-3-3η

**DOI:** 10.3390/cells9081827

**Published:** 2020-08-03

**Authors:** Hyun Jung Kwon, Duk-Soo Kim, Woosuk Kim, Hyo Young Jung, Yeon Hee Yu, Young In Ju, Dae-Kyoon Park, In Koo Hwang, Dae Won Kim, Dae Young Yoo

**Affiliations:** 1 Department of Biochemistry and Molecular Biology, Research Institute of Oral Sciences, College of Dentistry, Gangneung-Wonju National University, Gangneung 25457, Korea; donuts25@naver.com; 2 Department of Anatomy, College of Medicine, Soonchunhyang University, Cheonan-Si 31151, Korea; dskim@sch.ac.kr (D.-S.K.); yyh0220@sch.ac.kr (Y.H.Y.); nice125@sch.ac.kr (Y.I.J.); mdeornfl@sch.ac.kr (D.-K.P.); 3 Department of Biomedical Sciences and Research Institute for Bioscience and Biotechnology, Hallym University, Chuncheon 24252, Korea; tank3430@hallym.ac.kr; 4 Department of Anatomy and Cell Biology, College of Veterinary Medicine, and Research Institute for Veterinary Science, Seoul National University, Seoul 08826, Korea; hyoyoung@snu.ac.kr (H.Y.J.); vetmed2@snu.ac.kr (I.K.H.)

**Keywords:** cannabinoid receptor–interacting protein 1a, oxidative stress, ischemia, 14-3-3η

## Abstract

Cannabinoid receptor-interacting protein 1a (CRIP1a) binds to the *C*-terminal domain of cannabinoid 1 receptor (CB1R) and regulates CB1R activities. In this study, we made Tat-CRIP1a fusion proteins to enhance CRIP1a penetration into neurons and brain and to evaluate the function of CRIP1a in neuroprotection following oxidative stress in HT22 hippocampal cells and transient forebrain ischemia in gerbils. Purified exogenous Tat-CRIP1a was penetrated into HT22 cells in a time and concentration-dependent manner and prevented H_2_O_2_-induced reactive oxygen species formation, DNA fragmentation, and cell damage. Tat-CRIP1a fusion protein also ameliorated the reduction of 14-3-3η expression by H_2_O_2_ treatment in HT22 cells. Ischemia–reperfusion damage caused motor hyperactivity in the open field test of gerbils; however, the treatment of Tat-CRIP1a significantly reduced hyperactivity 1 day after ischemia. Four days after ischemia, the administration of Tat-CRIP1a restored the loss of pyramidal neurons and decreased reactive astrocytosis and microgliosis induced by ischemic damage in the hippocampal cornu Ammonis (CA)1 region. Ischemic damage decreased 14-3-3η expression in all hippocampal sub-regions 4 days after ischemia; however, the treatment of Tat-CRIP1 ameliorated the reduction of 14-3-3η expression. These results suggest that Tat-CRIP1a attenuates neuronal damage and hyperactivity induced by ischemic damage, and it restores normal expression levels of 14-3-3η protein in the hippocampus.

## 1. Introduction

Cerebral ischemia is one of the leading causes of death globally and results in a lower quality of life in survivors due to neurological disabilities [1,2]. Ischemia results in deficiencies in glucose and oxygen and leads to an ion imbalance and impairment of cellular homeostasis [3,4]. Excessive neuronal depolarization by ischemia causes excitotoxicity by releasing excitatory neurotransmitters, and an over-release of calcium ions activates deleterious enzymatic reactions, which can result in neuronal death [5]. Many recent studies have focused on various factors and materials to effectively inhibiting neuroinflammation and neuronal apoptosis following ischemia–reperfusion damage [6,7]. It is thought that understanding the molecular mechanisms of cerebral ischemia can advance therapeutic strategies for ischemic stroke [8].

The cannabinoid 1 receptor (CB1R) is a G protein-coupled receptor and is easily detected in the excitatory presynaptic regions in the hippocampus [9,10]. The cannabinoid receptor–interacting protein 1a (CRIP1a) binds to the *C*-terminal domain of CB1R and is involved in presynaptic neurotransmitter release by modulating calcium channel activity [11,12]. In vitro studies demonstrated that the overexpression of CRIP1a attenuated CB1R downregulation and inhibited CB1R signaling stimulated by the treatment of exogenous cannabinoid agonists [13]. Hippocampus-dependent memory and neural plasticity are modulated by cannabinoid signaling, but enhanced memory resulting from cannabinoid receptor activation does not follow a simple pattern [14]. The expression of CB1R and CRIP1a was significantly increased following excitotoxicity induced by kanic acid [15]. In addition, considerable evidence indicates that endogenous cannabinoid signaling is closely involved in neuroprotection, and these effects are induced by interactions of the cannabinoid with various transcription factors such as nuclear factor erythroid 2-related factor 2, nuclear Factor kappa-light-chain-enhancer of activated B cells, and peroxisome proliferator-activated receptors, which inhibit enormous neuroinflammation and oxidative stress in acute and chronic neurodegenerative diseases [16,17,18]. Recent studies have tried to treat neurodegenerative diseases such as Alzheimer’s and Parkinson’s diseases by cannabinoid targeting therapy [19,20,21].

The 14-3-3 proteins are abundantly expressed in the brain and involved in cellular signaling, proliferation, and processes of cell death [22]. It has been reported that 14-3-3 proteins interact with Bcl-2-associated X protein (BAX) and extracellular signal-regulated protein (ERK), which have crucial roles in apoptosis, and these pro-apoptotic proteins are upregulated during excitotoxicity-induced neurodegeneration. Unlike BAX and ERK, the expression of 14-3-3 proteins including 14-3-3η are downregulated during kainic acid-induced neurotoxicity [23]. On the other hand, in excitotoxicity-induced neuronal death, the expression of CB1R and CRIP1a was significantly increased [15], and decreased expression of 14-3-3η was restored during neuroprotection induced by exogenous cannabinoid treatment [24]. Actions of 14-3-3 proteins are closely related to cannabinoid receptor signaling [24,25], but the related mechanism between CRIP1a and 14-3-3 is not clear.

Roles of agonists and antagonists for CB1R in neuroprotection have been debated for the past decade; however, few studies have focused on the function of CRIP1a in mediated signaling CB1R. Understanding the exact roles of CRIP1a should precede an understanding of the mechanisms involved in cannabinoid signaling with respect to neuroprotection against ischemic damage. Therefore, in the present study, we investigated the expression of CRIP1a in the hippocampus after ischemia and examined the roles of CRIP1a in neuroprotection following hydrogen peroxide-induced cell damage in HT22 cells and in the gerbil hippocampus following 5 minutes of transient forebrain ischemia.

## 2. Materials and Methods

### 2.1. In Vitro Effects of Tat-CRIP1a in HT22 Cells

#### 2.1.1. Cell Preparation

Immortalized mouse hippocampal cells (HT22) were purchased from Sigma (St. Louis, MO, USA) and were maintained in Dulbecco’s modified eagle medium (DMEM) supplemented with 10% fetal bovine serum and antibiotics at 37 °C with humidified conditions of 95% air and 5% CO_2_ as described in a previous study [26].

#### 2.1.2. Construction of Expression Vectors

A cell-permeable Tat expression vector was prepared as described in a previous study [27]. Sense primer 5′-CTCGAGATGCGCCTCCGC-3′ and antisense primer 5′-GGATCCTTAGAGATCCTCCTGTGCC-3′ were used to amplify cDNA for CRIP1a by PCR. The PCR product was subcloned in a TA cloning vector (pGEM^®^-T easy vector; Promega Corporation, Madison, WI, USA) and ligated into the Tat expression vector to produce a Tat-His-CRIP1a fusion protein. The Tat domain consists of 9 amino acids, RKKRRQRRR, and is connected with a 6xHistidine tag. They are inserted in the *N*-terminal of CRIP1a. A His-CRIP1a (Control-CRIP1a) without the Tat domain was also prepared to use as a control.

The Tat-His-CRIP1a and His-CRIP1a-containing plasmids were transformed into *Escherichia coli* BL21 cells to produce both proteins. Isopropyl-β-d-thiogalactoside (0.1 mM, Duchefa, Haarlem, The Netherlands) was given to the bacterial cells at 18 °C for 8 h, and the harvested cells were purified with a Ni^2+^-nitrilotriacetic acid Sepharose affinity column and PD-10 column chromatography (Amersham, Braunschweig, Germany). To estimate the concentration of purified proteins, a Bradford assay was performed.

#### 2.1.3. Penetration Efficacy of Tat-CRIP1a Proteins in the HT22 Cells

The concentration and time-dependent intracellular penetration of purified exogenous His-CRIP1a and Tat-His-CRIP1a proteins were assessed following incubation with both proteins at various concentration (0.5–5 μM) for 60 min and 1 μM of both proteins at various time points (0–60 min) in HT22 cells, respectively. Then, the cells were treated with trypsin– ethylenediaminetetraacetic acid for 10 min and washed with phosphate-buffered saline (PBS) to eliminate proteins attached to the cellular membranes. Cells were lysed with ice-cold radioimmunoprecipitation assay buffer buffer (Thermo Scientific, Hanover Park, IL, USA) and Western blot was conducted using rabbit anti-polyhistidine primary antibody (1:2,000, His-probe, SantaCruz Biotechnology, Santa Cruz, CA, USA) or rabbit anti-CRIP1a antibody (1:1000, Novus Biologicals, Littleton, CO, USA) as described in a previous study [26]. In addition, the penetrated His-CRIP1a and Tat-His-CRIP1a proteins were visualized with immunocytochemical staining for polyhistidine after 1 μM of both proteins were incubated for 60 min with HT22 cells [26].

#### 2.1.4. Effects of Tat-CRIP1a Proteins on Cell Death and DNA Damage Exposed to H_2_O_2_ in the HT22 Cells

The neuroprotective effects of exogenous His-CRIP1a or Tat-His-CRIP1a against H_2_O_2_-induced oxidative damage were evaluated by water-soluble tetrazolium salt-1 (WST-1) and terminal deoxynucleotidyl transferase-mediated biotinylated deoxyuridine triphosphate nick end labeling (TUNEL) staining as described [26]. The WST-1 assay evaluates cell viability via the conversion of tetrazolium salts into formazans by the activity of cellular mitochondrial dehydrogenase. HT22 cells were treated with various concentrations of exogenous His-CRIP1a or Tat-His-CRIP1a proteins (0–1 μM) for 1 h, and oxidative damage was induced by incubation with 1 mM H_2_O_2_ for 5 h (WST-1 assay) and 3 h (TUNEL staining). Cell viability and DNA fragmentation were confirmed by WST-1 and TUNEL assay kits according to manufacturer’s protocol (Roche Diagnostics, Mannheim, Germany). In the WST-1 assay, HT22 cells were placed into 96-well plates at a concentration of 8 × 10^3^ cells/well. Cells were incubated for 24 h and 10 μL/well of WST-1 reagent was added to each well (1:10 dilution). HT22 cells were incubated with WST-1 reagent for 4 h in standard culture conditions. Optical density was measured for WST-1 assay at 450 nm using an ELISA microplate reader (Labsystems Multiskan MCC/340, Helsinki, Finland). TUNEL-positive fluorescence was obtained by a Fluoroskan ELISA plate reader (Labsystems Oy, Helsinki, Finland).

#### 2.1.5. Effects of Tat-CRIP1a Proteins on ROS Levels Exposed to H_2_O_2_ in the HT22 Cells

The formation of intracellular reactive oxygen species (ROS) was evaluated by the conversion of 2′,7′-dichlorofluorescein diacetate (DCF-DA) to DCF in HT22 cells as described previously [26]. The HT22 cells were incubated with 1 μM His-CRIP1a or Tat-His-CRIP1a proteins for 1 h and then sequentially treated with 1 mM H_2_O_2_ for 10 min and 20 μM DCF-DA for 30 min. DCF-positive fluorescence was quantified using a Fluoroskan ELISA plate reader (Labsystems Oy, Helsinki, Finland).

#### 2.1.6. Effects of Tat-CRIP1a Proteins on 14-3-3η Levels in the HT22 Cells

To elucidate the possible neuroprotective mechanisms of Tat-CRIP1a against H_2_O_2_-induced oxidative damage, HT22 cells were incubated with 1 μM His-CRIP1a or Tat-His-CRIP1a proteins for 1 h and then treated with 1 mM H_2_O_2_ for 3 h. Western blot was conducted using a rabbit anti-14-3-3η antibody (1:1000; Merck Millipore, Temecula, CA, USA) as described in a previous study [26].

### 2.2. Changes of CRIP1a after Ischemia and In Vivo Effects of Tat-CRIP1a against Ischemic Damage in Gerbils

#### 2.2.1. Experimental Animals

Male Mongolian gerbils were obtained from Japan SLC Inc. (Shizuoka, Japan). All animals were handled and cared for in accordance with the guidelines of current international laws and policies (National Institutes of Health Guide for the Care and Use of Laboratory Animals, Publication No. 85–23, 1985, revised 1996) to minimize physiological stress, and experimental procedures were approved by the Institutional Animal Care and Use Committee (IACUC) of Soonchunhyang University (SCH20-0007, approval date: 2020/03/04).

#### 2.2.2. Induction of Transient Forebrain Ischemia

Mongolian gerbils were anesthetized with a mixture of 2.5% isoflurane (Baxter, Deerfield, IL, USA) in 33% oxygen and 67% nitrous oxide. Both common carotid arteries were blocked with aneurysm clips for 5 min, as described in the previous study [28]. Body temperature was regulated at 37 ± 0.5 °C until recovery from anesthesia. Sham operation was performed without the occlusion of common carotid arteries for control animals.

#### 2.2.3. Experimental Design

To observe the temporal and spatial changes in CRIP1a immunoreactivity within the hippocampus, animals were sacrificed at 3 h, 6 h, 12 h, 1 day, 2 days, 4 days, and 7 days after ischemia (*n* = 5 in each time points). To confirm the penetration of Tat-His-CRIP1a into hippocampal sub-regions, a single injection of His-CRIP1a or Tat-His-CRIP1a was administered intraperitoneally to the animals, and they were sacrificed 8 h after administration as described in previous studies [29,30]. To elucidate the effects of Tat-CRIP1a in ischemic damage, experimental animals were divided into 4 groups: sham-operated control (sham) group, 10% glycerol-treated ischemic (vehicle) group, His-CRIP1a-treated ischemic (Control-CRIP1a) group, and Tat-His-CRIP1a-treated ischemic (Tat-CRIP1a) group. In the gerbils at 3 months of age (50–60 g body weight), transient forebrain ischemia was induced by the occlusion of both common carotid arteries for 5 min. Immediately after surgery, vehicle or the exogenous CRIP1a proteins were injected intraperitoneally (3 mg/kg).

#### 2.2.4. Open Field Test

To evaluate the effects of Tat-CRIP1a on hyperactivity induced by ischemic damage, an open field test was performed one day after ischemia/reperfusion, as described previously [26]. Motor activity was measured by distance traveled for 30 min and recorded with a digital camera system (Basler106200, Ahrensburg, Germany). The recorded data were reanalyzed by Ethovision XT14 (Noldus, Wageningen, The Netherlands).

#### 2.2.5. Tissue Processing and Immunohistochemistry

Animals were anesthetized with a mixture of alfaxalone (Alfaxan, 75 mg/kg; Careside, Seongnam, South Korea) and xylazine (10 mg/kg; Bayer Korea, Seoul, South Korea) at 3 h, 6 h, 12 h, 1 day, 2 days, 4 days, and 7 days after ischemia (*n* = 5 in each time points) and perfused transcardially with 0.1 M phosphate-buffered saline (PBS, pH 7.4) and 4% paraformaldehyde in 0.1 M PBS sequentially. The brains were removed and postfixed in 4% paraformaldehyde in 0.1 M PBS for 12 h at 25 °C. The brains were infiltrated with 30% sucrose overnight and cut into 30 μm tissue sections with a cryostat (Leica, Wetzlar, Germany). Sections between 1.4 and 2.0 mm posterior to the bregma were stained with rabbit anti-CRIP1a antibody (1:200, Novus Biologicals, Littleton, CO, USA), rabbit anti-polyhistidine antibody (1:200, His-probe, SantaCruz Biotechnology, Santa Cruz, CA, USA), 1% cresyl violet solution (Sigma, St. Louis, MO, USA), rabbit anti-glial fibrillary acidic protein (GFAP) antibody (1:1000; Merck Millipore, Temecula, CA, USA), rabbit anti-ionized calcium-binding adapter molecule 1 (Iba-1) antibody (1:500; Wako, Osaka, Japan), or rabbit anti-14-3-3η antibody (1:1000; Merck Millipore) according to the previous study [26].

#### 2.2.6. Semi-Quantification of Data

The immunoreactivity of CRIP1a and 14-3-3η was analyzed using ImageJ software v. 1.5 (National Institutes of Health, Bethesda, MD, USA). Digital images of stained hippocampi were captured using a BX51 light microscope (Olympus, Tokyo, Japan) with a digital camera (DP72, Olympus) and calibrated into 512 × 512 pixels. Each pixel had 256 gray levels, and the intensity of each picture was represented by relative optical density (ROD), which was the transformed mean gray level by the formula: ROD = log (256/mean gray level). ROD of the background staining was subtracted to correct for nonspecific staining, using ImageJ v. 1.50 software (National Institutes of Health). Data are expressed as a percentage of the sham groups.

Cresyl violet-positive neurons were taken from the midpoint of the hippocampal CA1 region from four sections at 120 µm intervals, and the number of cresyl violet-positive cells was counted using OPTIMAS software (version 6.5; CyberMetrics^®^ Corporation, Phoenix, AZ, USA). Data are calibrated into percentile values versus control group.

#### 2.2.7. Statistical Analysis

The data were expressed as the mean of the experiments performed for each experimental investigation. To evaluate the changes and effects of Tat-CRIP1a after ischemic damage, the differences among the means were statistically analyzed using a one-way or two-way analysis of variance (ANOVA), followed by Bonferroni’s post-hoc test with GraphPad Prism 5.01 software (GraphPad Software, Inc., La Jolla, CA, USA). The results were considered statistically significant when *p* < 0.05.

## 3. Results

### 3.1. In Vitro Effects of Tat-CRIP1a in HT22 Cells

#### 3.1.1. Expression and Purification of Tat-CRIP1a Protein

**Figure 1:**
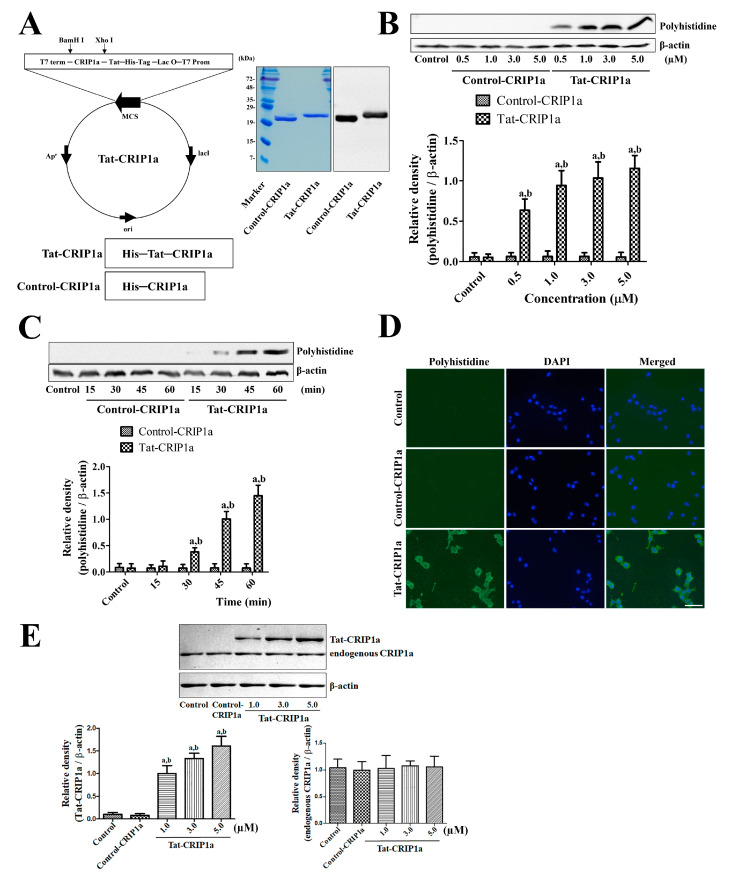
Construction of the control and Tat-CRIP1a fusion proteins and confirmation of efficient delivery into HT22 cells. (**A**) Expression and purification of the His-CRIP1a and Tat-His-CRIP1a proteins were confirmed by Coomassie brilliant blue staining and Western blot analysis with an anti-polyhistidine antibody. (**B**) The delivery of exogenous His-CRIP1a and Tat-His-CRIP1a proteins in various concentrations was evaluated by Western blot for polyhistidine at 60 min after the treatment. (**C**) Time-dependent delivery of 1 μM His-CRIP1a and Tat-His-CRIP1a proteins was confirmed by Western blot for polyhistidine. Bar graph represents the mean with standard deviation. (**B**) and (**C**) Density of polyhistidine and β-actin bands were analyzed and the data were calibrated using a ratio of polyhistidine/β-actin. A two-way ANOVA test was used to analyze the data followed by a Bonferroni’s post-hoc test (^a^ *p* < 0.05, significantly different from the Control-CRIP1a group; ^b^ *p* < 0.05, significantly different from the Control group). Data are expressed as mean with standard deviation. (**D**) Visualization of internalized His-CRIP1a and Tat-His-CRIP1a proteins with immunocytochemical staining for polyhistidine at 60 min after treatment. Scale bar = 50 μm. (**E**) Penetration of exogenous Tat-CRIP1a and expression of endogenous CRIP1a proteins are confirmed by Western blot analysis with an anti-CRIP1a antibody. Data were expressed by ratio of Tat-CRIP1a/β-actin or endogenous CRIP1/β-actin. One-way ANOVA test was used to analyze the data followed by a Bonferroni’s post-hoc test (^a^ *p* < 0.05, significantly different from the Control-CRIP1a group; ^b^ *p* < 0.05, significantly different from the Control group). CRIP1a: cannabinoid receptor–interacting protein 1a, Tat-CRIP1a: Tat-His-CRIP1a-treated ischemic.

To generate a Tat-His-PDIA3 fusion protein, the human CRIP1a gene was fused to a Tat peptide expression vector, and the His-CRIP1a protein was manufactured without a Tat domain. Purified proteins were separated with confirmation by Western blot analysis. Prominent Tat-His-CRIP1 protein and His-CRIP1a protein bands were found with about 1.6–1.7 kDa differences, which is the molecular weight for the Tat peptide, on Western blot using rabbit anti-polyhistidine antibody (Figure 1A).

#### 3.1.2. In Vitro Efficacy of Intracellular Delivery of Tat-CRIP1a Protein in HT22 Cells

The concentration and time-dependent intracellular delivery of purified exogenous His-CRIP1a and Tat-His-CRIP1a was determined by Western blot analysis using a polyhistidine antibody. Polyhistidine bands were not observed in His-CRIP1a-treated cells at any concentration (0.5–5.0 μM) or time after treatment. In contrast, incubation with Tat-CRIP1a resulted in strong polyhistidine bands, and their density increased in a concentration and time-dependent manner (Figure 1B,C). Significant increases of polyhistidine levels were found at 0.5 μM and 30 min after CRIP1a treatment. The intracellular delivery of exogenous His-CRIP1a and Tat-His-CRIP1a was confirmed using immunofluorescent staining for polyhistidine in HT22 cells. In the vehicle-treated control and Control-CRIP1a-treated groups, no polyhistidine immunoreactive structures were detected in HT22 cells. However, in the Tat-CRIP1a-treated group, strong polyhistidine immunoreactivity was found in the cytoplasm of HT22 cells (Figure 1D). Endogenous and exogenous CRIP1a were detected by Western blot analysis using a CRIP1a antibody. In the Tat-CRIP1a-treated groups, we observed double bands, which indicate endogenous and exogenous CRIP1a, respectively, and density of the bands for Tat-CRIP1a increased with concentration dependently. In the groups without exogenous Tat-His-CRIP1a treatment, we only detected the bands of endogenous CRIP1a (Figure 1E).

#### 3.1.3. Effects of Tat-CRIP1a Proteins on Cell Death, DNA Damage, ROS, and 14-3-3η Levels Following Exposure to H_2_O_2_

**Figure 2:**
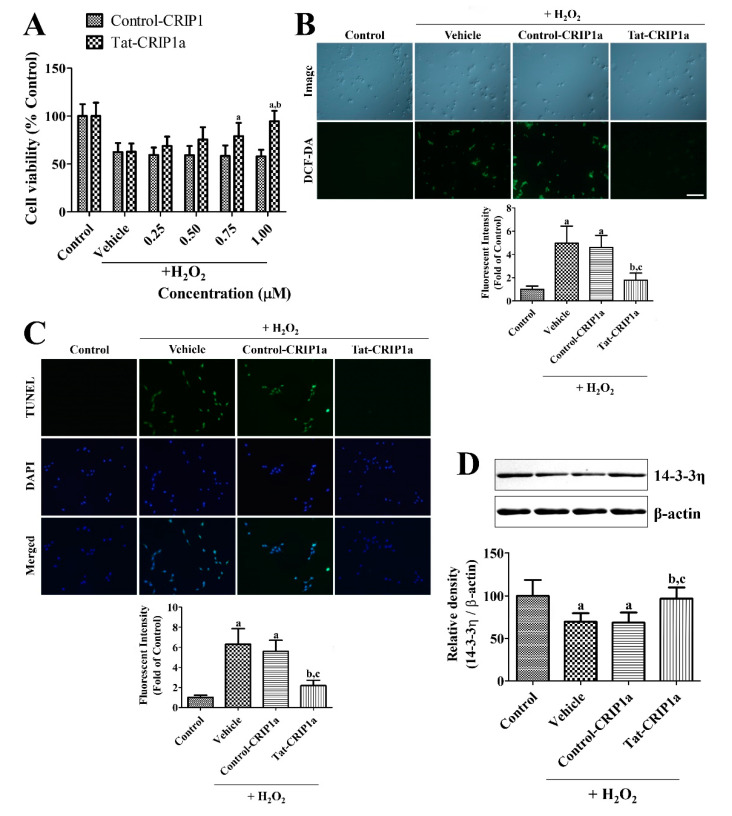
In vitro neuroprotective effects and mechanism of His-CRIP1a and Tat-His-CRIP1a proteins against H_2_O_2_-induced damage in HT22 cells. (**A**) Concentration-dependent cell viability was observed in HT22 cells after His-CRIP1a and Tat-His-CRIP1a treatment for 1 h and subsequent treatment with 1 mM H_2_O_2_ for 5 h. A two-way ANOVA test was used to analyze the data followed by a Bonferroni’s post-hoc test (^a^ *p* < 0.05, significantly different from the Control-CRIP1a group; ^b^ *p* < 0.05, significantly different from the vehicle group). (**B**) H_2_O_2_-induced reactive oxygen species (ROS) production was measured with 1.0 μM His-CRIP1a and Tat-His-CRIP1a treatment for 60 min and subsequent treatment with 1 mM H_2_O_2_ for 10 min and 20 μM 2′,7′-dichlorofluorescein diacetate (DCF-DA) for 30 min. (**C**) H_2_O_2_-induced DNA fragmentation was visualized with TUNEL staining after 1 μM His-CRIP1a and Tat-His-CRIP1a treatment for 1 h and subsequent treatment with 1 mM H_2_O_2_ for 3 h. (**B** and **C**) Scale bar = 100 μm. Intensities of DCF-stained and terminal deoxynucleotidyl transferase-mediated biotinylated dUTP nick end labeling (TUNEL)-positive cells were measured and the data are presented as percentile values vs. Control group. (**D**) Levels of 14-3-3η were measured with Western blot after 1 μM His-CRIP1a or Tat-His-CRIP1a proteins for 1 h and then treated with 1 mM H_2_O_2_ for 3 h. The density of 14-3-3η and β-actin bands were analyzed, and the data was calibrated with a ratio of 14-3-3η/β-actin. A one-way ANOVA test was used to analyze the data followed by a Bonferroni’s post-hoc test (^a^ *p* < 0.05, significantly different from the control group; ^b^ *p* < 0.05, significantly different from the vehicle group; ^c^ *p* < 0.05, significantly different from the Control-CRIP1a group). Data are expressed as mean with standard deviation.

In the vehicle-treated group, cell viability was significantly decreased to 62.2% of the control group after H_2_O_2_ exposure (Figure 2A). In the Control-CRIP1a-treated groups, cell viability was observed as 57.6–59.0% of the control group and did not show any significant changes depending on the concentration of His-CRIP1a. In contrast, incubation with Tat-CRIP1a showed significant increases in cell viability with values of 68.4–94.2% compared to the control group and changed in a concentration-dependent manner.

In the control group, very little DCF fluorescence was detected in the HT22 cells. In the vehicle-treated group, cells with strong DCF staining were found after H_2_O_2_ exposure, and fluorescence intensity was dramatically increased by 496.9% compared to the control group. In the His-CRIP1a-treated cells, DCF-stained cells were found in abundance after H_2_O_2_ exposure, and a fluorescence intensity similar to the vehicle-treated group was found. In the Tat-His-CRIP1a-treated group, few DCF-stained cells were observed after H_2_O_2_ exposure, and the fluorescence intensity was significantly decreased by 178.4% compared to the control group vehicle or Control-CRIP1a-treated group (Figure 2B).

In the control group, few TUNEL-positive cells were found in the HT22 cells. In the vehicle and Control-CRIP1a-treated groups, many TUNEL-positive cells were found in the HT22 cells after H_2_O_2_ exposure, and fluorescence intensity was significantly increased by 630.2% and 560.7% compared to the control group, respectively. In the Tat-CRIP1a-treated group, few TUNEL-positive cells were found in the HT22 cells after H_2_O_2_ exposure, and the fluorescence intensity was significantly decreased to 218.2% of the control group compared to that of the vehicle or Control-CRIP1a-treated group (Figure 2C).

In the vehicle group, 14-3-3η levels were significantly decreased to 69.8% of the control group. In the Control-CRIP1a group, similar levels of 14-3-3η was observed compared to that of the vehicle group. In the Tat-CRIP1a group, 14-3-3η levels were significantly increased compared to that in the vehicle or Control-CRIP1a group with similar results for the Control group (Figure 2D).

### 3.2. Changes of CRIP1a after Ischemia and the In Vivo Effects of Tat-CRIP1a Following Ischemic Damage in Gerbils

#### 3.2.1. Changes of CRIP1a Immunoreactivity in the Hippocampal CA1 Region after Transient Forebrain Ischemia

**Figure 3:**
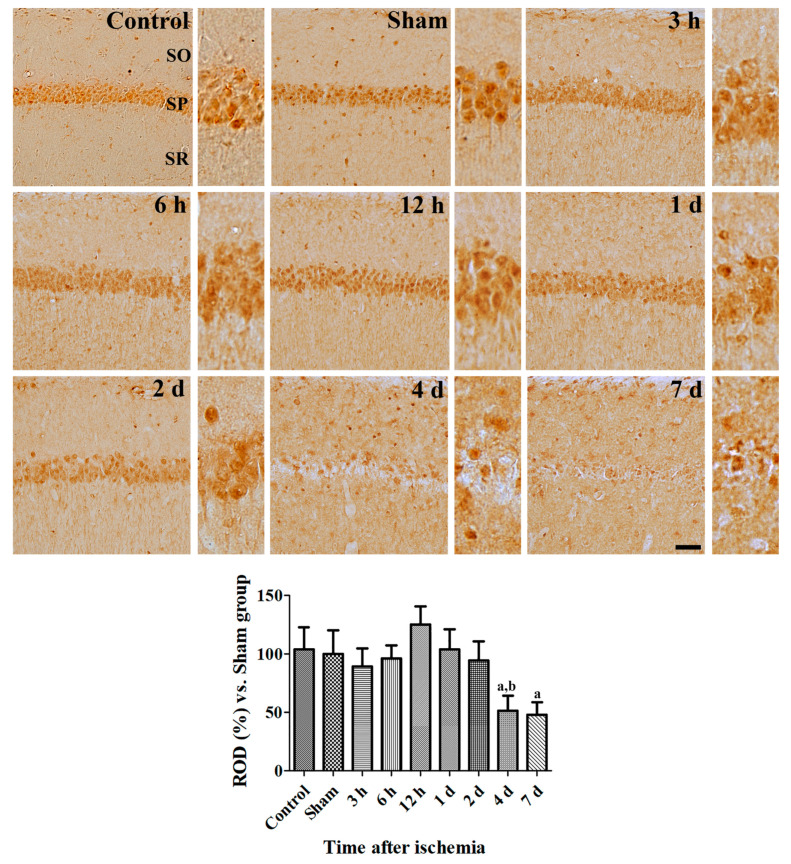
Spatial and temporal changes of CRIP1a immunoreactivity in the gerbils at various time points after ischemia. Relative optical densities in each region are described as a percentage of the value of the sham group, and a one-way ANOVA test was used to analyze the data followed by Bonferroni’s post-hoc test (*n* = 5 per group; ^a^ *p* < 0.05, significantly different from the Sham group; ^b^ *p* < 0.05, significantly different from the pre-adjacent group). SO, stratum oriens; SP, stratum pyramidale; SR, stratum radiatum.

In the sham-operated group, CRIP1a immunoreactivity was found in non-pyramidal and pyramidal cells in the CA1 region. From 3 to 12 h after ischemia/reperfusion, similar CRIP1a immunoreactivity was found in the control group, but fewer CRIP1a immunoreactive cells were detected in the non-pyramidal cells located in the stratum oriens and radiatum. One to two days after ischemia, fewer CRIP1a immunoreactive structures were seen in the stratum pyramidale of the CA1 region. In addition, CRIP1a immunoreactivity was significantly decreased in the hippocampal CA1 region 2 days after ischemia compared to that in the sham-operated group. Four and seven days after ischemia, CRIP1a immunoreactive structures were only found in the non-pyramidal cells because of neuronal death in pyramidal cells (Figure 3).

#### 3.2.2. Effects of CRIP1a on Ischemia-Induced Motor Activity and Cell Death

**Figure 4:**
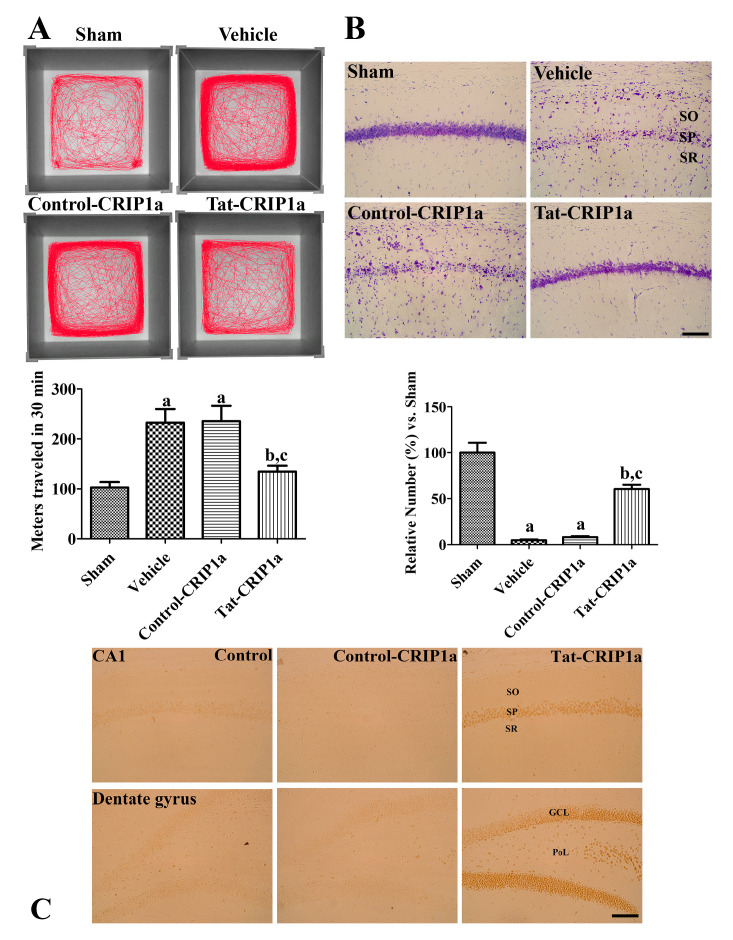
In vivo neuroprotective effects of Control-CRIP1a and Tat-CRIP1a proteins against ischemic damage in gerbils. (**A**) Images showing track visualization for 30 min in each groups 1 day after ischemia. (**B**) Cresyl violet staining in the hippocampal CA1 region of the sham, vehicle, Control-CRIP1a, and Tat-CRIP1a groups at 4 days after ischemia reperfusion. (**C**) Confirmation of penetrated Tat-CRIP1a into neurons in the SP, GCL and PoL in the Tat-CRIP1a group using anti-histidine antibody. A single injection of His-CRIP1a or Tat-His-CRIP1a was administered intraperitoneally to the animals, and they were sacrificed 8 h after administration. Scale bar = 100 µm. SO, stratum oriens; SP, stratum pyramidale; SR, stratum radiatum; GCL, granule cell layer; PoL, polymorphic layer. Meters moved in 30 min, described as mean values, and the number of cresyl violet-positive cells are shown as a percentage of the value of the sham group. A one-way ANOVA test was used to analyze the data followed by Bonferroni’s post-hoc test (*n* = 5 per group; ^a^ *p* < 0.05, significantly different from the control group; ^b^ *p* < 0.05, significantly different from the vehicle group; ^c^ *p* < 0.05, significantly different from the Control-CRIP1a group). Data are expressed as mean with standard deviation.

Spontaneous motor activity was assessed by the open field test to evaluate hyperactivity after ischemic damage. In the sham group, the mean distance traveled in 30 min was 102.7 m. In the vehicle and Control-CRIP1a groups, spontaneous motor activity was significantly increased compared to the sham group (232.7 and 235.5 m for 30 min respectively). In contrast, the administration of Tat-His-CRIP1a significantly decreased motor hyperactivity (134.6 m) compared to the vehicle group (Figure 4A).

To evaluate the effects of Tat-CRIP1a proteins on neuroprotection following transient forebrain ischemia, cresyl violet staining was performed on hippocampal sections 4 days after ischemia. In the CA1 region of the sham group, abundant cresyl violet positive cells were mainly detected in the stratum pyramidale. In the vehicle and Control-CRIP1a groups, the number of cresyl violet positive cells were significantly decreased (4.9% and 8.3% of the sham group, respectively). In the Tat-CRIP1a group, the number of cresyl violet-positive cells was significantly increased (60.1% of control group) compared to that of the vehicle group (Figure 4B).

To confirm the penetration of exogenous Tat-His-CRIP1a into hippocampal neurons, immunohistochemistry for histidine was performed. In the control and Control-CRIP1a groups, histidine immunoreactivity was rarely detected in the dentate gyrus and CA 1 region. In the Tat-CRIP1a group, strong histidine immunoreactivity was detected in the neurons of the stratum pyramidale, granule cell layer, and polymorphic layer (Figure 4C).

#### 3.2.3. Roles of Tat-CRIP1a on Glial Activation in Hippocampal CA1 Region

**Figure 5:**
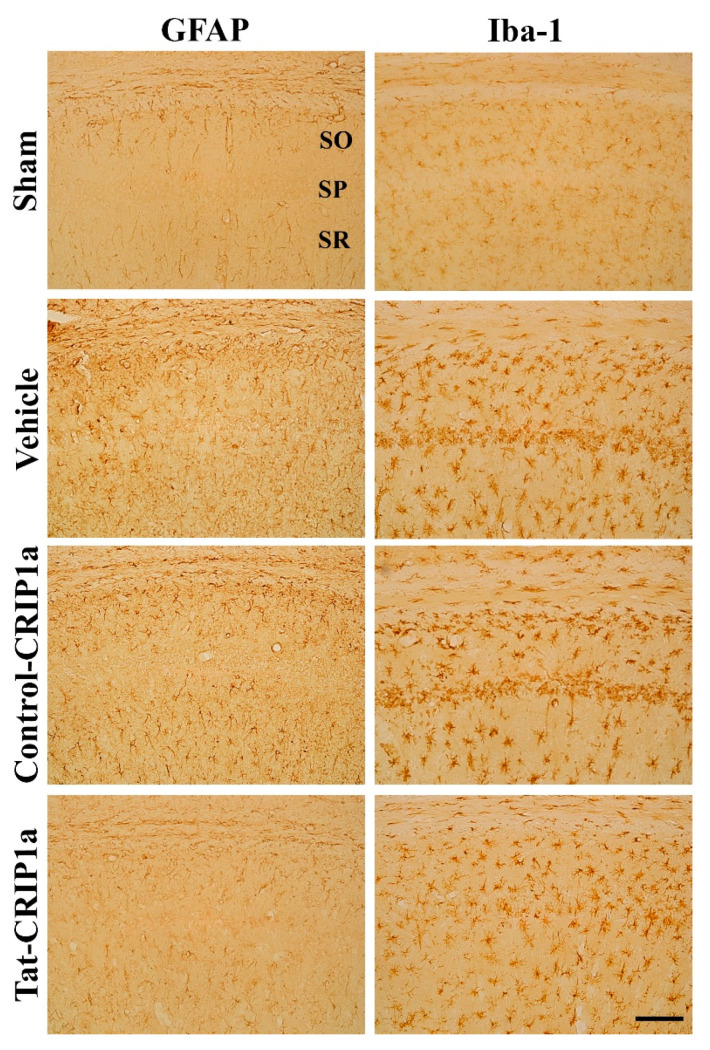
Immunohistochemistry for glial fibrillary acidic protein (GFAP) and Iba-1 in the cornu Ammonis (CA)1 region of the sham, vehicle, Control-CRIP1a, and Tat-CRIP1a groups at 4 days after ischemia. In the vehicle group, GFAP-immunoreactive astrocytes have hypertrophied cytoplasm and processes and ionized calcium-binding adapter molecule 1 (Iba-1)-imunoreactive microglia with hypertrophied cytoplasm with retracted processes. In the Tat-CRIP1a group, astrocytes and microglia with activated phenotypes were markedly decreased. Scale bar = 100 µm. SO, stratum oriens; SP, stratum pyramidale; SR, stratum radiatum.

In the sham group, GFAP-positive astrocytes and Iba1-positive microglia had small amounts of cytoplasm and thin processes in the CA1 region of the hippocampus. In the vehicle and Control-CRIP1a groups, GFAP-positive astrocytes exhibited hypertrophic cytoplasm 4 days after ischemia. In addition, Iba1-positive microglia had large cell bodies and thick processes in the stratum oriens and radiatum, while Iba-1-positive microglia showed a phagocytic phenotype in the stratum pyramidale where neuronal death occurred after transient forebrain ischemia. In the Tat-CRIP1a group, the morphology of astrocytes and microglia was similar to that of the sham group; however, some astrocytes and microglia exhibited large amounts of cytoplasm and thick processes (Figure 5).

#### 3.2.4. Effects of Tat-PDIA3 on 14-3-3-eta Expression in the Hippocampal Sub-Regions

**Figure 6:**
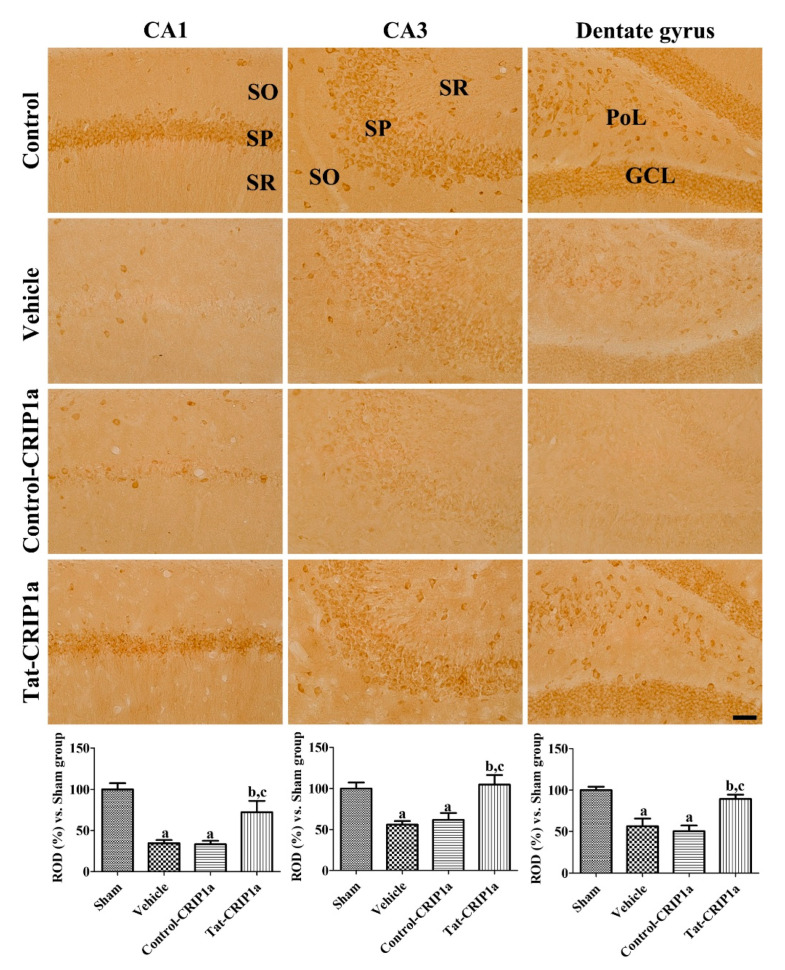
Immunohistochemistry for 14-3-3η in the hippocampal sub-regions of the sham, vehicle, Control-CRIP1a, and Tat-CRIP1a groups at 4 days after ischemia. In the CA1 and CA3 regions, the immunoreactivity of 14-3-3η was primarily detected in pyramidal neurons. In the dentate gyrus, the immunoreactivity of 14-3-3η was observed in the polymorphic layer and granule cell layer. Scale bar = 100 µm. SO, stratum oriens; SP, stratum pyramidale; SR, stratum radiatum; PoL, polymorphic layer; GCL, granule cell layer. Relative optical densities in each region are described as a percentage of the value of the sham group, and a one-way ANOVA test was used to analyze the data followed by a Bonferroni’s post-hoc test (*n* = 5 per group; ^a^ *p* < 0.05, significantly different from the control group; ^b^ *p* < 0.05, significantly different from the vehicle group; ^c^ *p* < 0.05, significantly different from the Control-CRIP1a group).

In the sham group, 14-3-3η immunoreactivity was mainly detected in the pyramidal cells of the CA1 and CA3 regions as well as in granule cells and polymorphic layers of the dentate gyrus. In the vehicle and Control-CRIP1a groups, the immunoreactivity of 14-3-3η was significantly decreased in all regions of the hippocampus compared to those in the sham group. In the Tat-CRIP1a group, 14-3-3η immunoreactive structures were abundant in the hippocampal CA1, CA3, and dentate gyrus. In this group, 14-3-3η immunoreactivity was significantly higher in these regions than in the vehicle and Control-CRIP1a groups and was 72.4%, 104.9%, and 89.4% of the control group in the CA1, CA3, and dentate gyrus, respectively (Figure 6).

## 4. Discussion

Cannabinoid signaling is closely involved in neuroprotection through various mechanisms [18,31], but their exact actions do not follow a simple pattern, and the processes involved remain controversial. It has recently been identified that CRIP1a binds to the *C*-terminal of CB1R in the presynaptic terminal to modulate its activity [11], and both CRIP1a and CB1R expression are increased by treatment with an excitotoxin [15]. In the present study, we evaluated the neuroprotective role of CRIP1a against H_2_O_2_-induced oxidative stress in HT22 cells and ischemia-induced neuronal damage in the gerbil hippocampus. First, we made a Tat-CRIP1a protein to facilitate the delivery of the protein into brain tissue and neurons because the Tat peptide acts as a transfection carrier of proteins and delivers the Tat-CRIP1a fusion protein into cells [32,33]. The purified exogenous Tat-His-CRIP1a easily penetrated into HT22 cells in a time and concentration-dependent manner, and treatment with Tat-CRIP1a, but not His-CRIP1a, significantly ameliorated H_2_O_2_-induced ROS formation and neuronal damage in HT22 cells. CRIP1a has shown responsiveness to both CB1R agonists and antagonists [34], which have neuroprotective effects following excitoxicity [35,36].

We also confirmed that the treatment of CRIP1a mitigated neuronal loss and a hyperactive motor phenotype induced by ischemic damage. It has been reported that the expression of CRIP1a mRNA is co-localized with CB1R mRNA in hippocampal pyramidal neurons and interneurons [12]. Following ischemic damage, deficiencies in oxygen and glucose results in excitotoxicity through the massive release of excitatory neurotransmitters, such as glutamate, which can result in neuronal death [37]. Presynaptic calcium entry is necessary for neurotransmitter release, and it is well known that CB1R activity inhibits presynaptic calcium channels. CRIP1a interacts with CB1R, suppressing the internalization of CB1R, which is essential for limiting glutamate release into the synaptic cleft [38]. The inhibition of internalization is caused by competing for β-arrestin [39,40], and it has been also reported that CRIP1a delivers newly synthesized CB1Rs to the presynaptic membrane without exogenous agonists of CB1R [41]. In hippocampal pyramidal neurons, CRIP1a overexpression prolongs the inhibition of excitatory currents induced by cannabinoids and decreases the severity of seizure [12], suggesting that treatment with CRIP1a may reduce ischemic neuronal damage by attenuating excitotoxicity in the present study.

In this study, we observed a decreased expression of 14-3-3η protein in HT22 cells and in gerbil hippocampal sub-regions 4 days following ischemia. The 14-3-3 family proteins are involved in many biological processes such as cell survival, proliferation, apoptosis, and gene expression. The deletion of all 14-3-3 isoforms in mice results in death [24,42]. The 14-3-3 proteins exhibit anti-apoptotic effects through interactions with Bcl-2-associated agonist of cell death (BAD), BAX, and p53, which are well-known pro-apoptotic proteins [43]. Moreover, an altered expression and function of the 14-3-3 proteins have been detected in neurodegenerative diseases such as Alzheimer’s disease and Parkinson’s disease [44,45]. Treatment with kainic acid, which causes excitotoxic neuronal loss, resulted in a decreased 14-3-3η expression and increased BAX in cortical neurons [23]. In cardiomyocytes, 14-3-3η expression is decreased after anoxia and anoxia/reperfusion [46]. It has also been reported that 14-3-3η is involved in cannabinoid signaling, and expression following excitotoxin was reversed by treatment with the CB1R agonist delta-9-tetrahydrocannabinol in another study [24]. In the present study, we also observed that treatment with CRIP1a reversed the decrease in 14-3-3η expression induced by forebrain ischemia. CRIP1a has been reported to modulate cyclic adenosine 3′, 5′-monophosphate (cAMP) production, and expression of the 14-3-3η protein is regulated by cAMP and other cellular signals [40,47]. These results support that the expression level of 14-3-3η is closely associated with neuronal survival following forebrain ischemia and can be changed by the action of CRIP1a.

In conclusion, we observed that the treatment of Tat-CRIP1a attenuated neuronal damage and normalized expression of the 14-3-3 η protein in hippocampal sub-regions. These results suggest that CRIP1a and its related cannabinoid molecules could be considered a therapeutic target for attenuating neuronal apoptosis following ischemic damage.
